# Peri-Implant Soft Tissue in Contact with Zirconium/Titanium Abutments from Histological and Biological Perspectives: A Concise Review

**DOI:** 10.3390/cells14020129

**Published:** 2025-01-17

**Authors:** Fatma A. N. Abouel Maaty, Mai A. Ragab, Yasmin M. El-Ghazawy, Fatma I. Elfaiedi, Marwa M. S. Abbass, Israa Ahmed Radwan, Dina Rady, Sara El Moshy, Nahed Sedky Korany, Geraldine M. Ahmed, Christof E. Dörfer, Karim M. Fawzy El-Sayed

**Affiliations:** 1 Oral Biology Department, Faculty of Dentistry, Cairo University, Cairo 12613, Egypt; fatma_nasser@dentistry.cu.edu.eg (F.A.N.A.M.); marwa.magdy@dentistry.cu.edu.eg (M.M.S.A.); esraa.ahmed@dentistry.cu.edu.eg (I.A.R.); dina.radi@dentistry.cu.edu.eg (D.R.); sarah.mahmoud@dentistry.cu.edu.eg (S.E.M.); nahed.korany@dentistry.cu.edu.eg (N.S.K.); 2 Oral Biology Department, Faculty of Dentistry, Ahram Canadian University, 6th of October City 12451, Egypt; mai_ali@dentistry.cu.edu.eg; 3 Department of Oral Biology, Faculty of Oral and Dental Medicine, Future University, Cairo 11835, Egypt; yasmin.elghazawy@dentistry.cu.edu.eg; 4 Oral Biology Department, Faculty of Dentistry, Sirte University, Sirte 674, Libya; fatma-ibrahim@dentistry.cu.edu.eg; 5 Stem Cells and Tissue Engineering Research Group, Faculty of Dentistry, Cairo University, Cairo 11835, Egypt; geraldine.ahmed@dentistry.cu.edu.eg; 6 Department of Endodontics, Faculty of Dentistry, Cairo University, Cairo 11835, Egypt; 7 Clinic for Conservative Dentistry and Periodontology, School of Dental Medicine, Christian Albrechts University, 24118 Kiel, Germany; doerfer@konspar.uni-kiel.de; 8 Oral Medicine and Periodontology Department, Faculty of Dentistry, Cairo University, Cairo 11835, Egypt

**Keywords:** dental implants, abutments, peri-implant soft tissue, zirconium, titanium, histological evaluation, biocompatibility

## Abstract

Dental implants are crucial in contemporary oral rehabilitation, necessitating optimal integration with the surrounding soft tissues for durable success. The attachment between the implant surface and peri-implant mucosa should establish a secure seal to prevent bacterial infiltration and subsequent tissue inflammation. This concise review examines the histological and biological perspectives of peri-implant soft tissue reactions to zirconium and titanium abutments, shedding light on their respective advantages and limitations. While titanium has been the gold standard, zirconia has gained attention due to its biocompatibility and aesthetic appeal. Histological studies show comparable soft tissue attachment and inflammatory responses between the two materials. Further research is needed to explore surface treatments and optimize outcomes in dental implant rehabilitation.

## 1. Introduction

Dental implants have developed a vital aspect of contemporary dental restoration practices. Besides the osseointegration of the implant into the alveolar bone, the integration with the surrounding soft tissues is an essential factor for the temporal stability of dental implants under loading. It is essential to have contact between the implant and the surrounding soft tissue to establish protective barriers against infectious organisms that could colonize the underlying tissues and cause peri-implantitis [1].

Soft tissues around implant abutments are functionally divided into a lining and masticatory mucosa. Masticatory mucosa is further divided into free and attached gingiva. What provides the intimate firmness of the mucosal seal around the abutment and prevents its detachment is the attached gingiva. Unfortunately, the attached gingiva–abutment interface is considered weaker than the surrounding natural tooth representing a critical site [2].

For instance, over time, dental implant manufacturers have improved dental implant abutment by incorporating surface treatments like sandblasting and acid etching, resulting in either smoothened surfaces or roughened surfaces. These modifications are almost effective in enhancing osseointegration by expanding the contact between bone cells and the implant surface but without obvious influence on the surrounding soft tissue [3]. After this, attention has focused on probable changes in the implant surface where it is in contact with soft tissues. The connective tissue surrounding the transmucosal portion of the implants helps prevent the epithelial tissues’ downward pull, which consequently greatly lowers the hazard of peri-implant mucositis and peri-implantitis [4].

About 60% of patients have experienced bacterial colonization on their implant surfaces. This is thought to be among the main reasons for both early and delayed implant failure, as it can trigger inflammatory processes in the host’s soft tissues and bone [5]. As a result of plaques caused by pathogenic bacteria infiltrating peri-implant sulcus, peri-implantitis represents one of the key reasons for dental implant malfunction [6]. Along with a systematic review, the occurrence of peri-implantitis varies from 0.4% to 43.9% within 5 years, and prevalence ranges from 1.1% to 85% [7].

Therefore, the ideal implant abutment material should be able to maintain longstanding homeostasis in the peri-implant mucosal environment. The trans-mucosal portion of abutments must be biocompatible to prevent causing inflammation in the peri-implant tissues. Apart from recent advances in dental abutment materials [8], the most used dental implant abutment materials remain titanium and zirconia [9], with titanium being considered the gold standard. Nevertheless, it has been criticized for many inherent weaknesses, including reported hypersensitivity reactions, biocompatibility issues, and unaesthetic gray color [10]. Thus, zirconia was introduced as an aesthetic alternative to titanium, showing biocompatibility, good tissue integration, less plaque accumulation, and subsequently low susceptibility to biofilm formation [11].

From a clinical standpoint, the available literature features a limited number of controlled clinical trials/randomized clinical trials (RCTs) that compare titanium implants to zirconia implants, which hinders the ability to reach definitive conclusions. Despite the trend indicating a higher failure rate for zirconia implants, the results do not show a better survival rate for titanium implants when compared to zirconia implants in the short term (12 months). While zirconia implants may provide aesthetic benefits, the few RCTs available in the literature do not indicate any clear advantages [12]. A recent systematic review and meta-analysis of RCTs found that titanium dental implants show a higher survival rate and less marginal bone loss compared to zirconia dental implants after one year of follow-up. Future RCTs that consider confounding factors and evaluate clinical, radiographic, and patient-reported outcomes are essential to establish the long-term efficacy and success of zirconia implants [13].

To date, a comprehensive review highlighting the peri-implant tissue response to titanium and zirconia implant surfaces and their modifications from a biological perspective is lacking. Therefore, this review was conducted based on studies that elaborate on histological and immunohistochemical analyses, as well as gene and protein expression, following the use of titanium and zirconia implants.

## 2. Research Methodology

**Figure 1:**
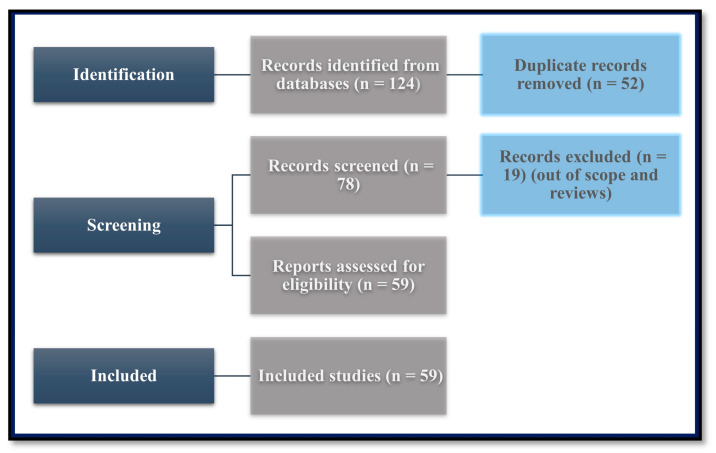
PRISMA flow diagram illustrating study selection methodology. Initial database search yielded 124 records, with subsequent filtering through duplicate removal (*n* = 52), preliminary screening (*n* = 78), and eligibility assessment resulting in 59 studies meeting final inclusion criteria.

This narrative review followed a structured approach to identify, screen, and select relevant studies addressing the impact of titanium and zirconium abutments on peri-implant soft tissues from a histological and biological perspective. The methodology was divided into the following steps (Figure 1).

### 2.1. Identification

Studies were identified through comprehensive searches of multiple electronic databases, including PubMed, Cochrane Library, and Google Scholar. Keywords used in the search strategy included “dental implants”, “implant abutment”, “titanium abutments”, “zirconium abutments”, “peri-implant soft tissues”, “transmucosal surface”, “histological”, “immunohistochemical”, “molecular analysis”, “molecular mechanism”, and “gene expression”. A total of 124 records were retrieved from these sources.

### 2.2. Removal of Duplicates

Duplicate records were identified and removed using reference management software. A total of 52 duplicate records were excluded at this stage.

### 2.3. Screening

The remaining 78 records were screened based on their titles and abstracts to assess their relevance to the research topic. During this phase, 19 records were excluded as they did not meet the inclusion criteria or were out of scope.

### 2.4. Eligibility Assessment

A total of 59 full-text articles were assessed for eligibility. Studies were evaluated based on their focus on histological, immunohistochemical, and molecular outcomes related to titanium and zirconium abutments. Exclusion criteria included studies unrelated to the research topic, insufficient data on soft tissue outcomes, or those that lacked relevant experimental or clinical results.

### 2.5. Inclusion

After a detailed assessment, 59 studies were included in the final analysis.

## 3. Histological Evaluation

### 3.1. Human Studies

Histologic investigations may provide crucial information about each material’s potential to form and seal soft tissues around implants in the best possible way. These histological investigations on the soft tissues around implants are typically carried out with soft tissue punches that are specifically made to allow for the elimination of soft tissue as one single bounded entity [14].

In an RCT, histological analyses for the total attachment length (evaluated from the highest part of the epithelial attachment to the lowest part of the connective tissue) and the inflammatory response in terms of soft tissues’ semi-quantitative assessment of the quantity and distribution of inflammatory cells nearby titanium, zirconia, and gold alloy abutment materials for patients requiring implant implantation in the posterior part of the mandible or maxilla were performed. A biopsy was carried out, and the abutments were detached with a layer of soft tissues to be examined eight weeks after surgery. Non-statistically significant differences were found in the inflammatory response and soft tissue attachment between zirconia and titanium abutments [15]. Additionally, a study has been conducted to investigate the soft tissue health of patients who had two implants in the lower arch with either titanium or zirconia abutments. There were no appreciable variations in the vascular intensity of the tissues near zirconia or titanium abutment surfaces after three months. The stratified squamous epithelium in both abutments was markedly keratinized and unceasing with respect to the junctional epithelium fronting the abutment surface [16].

Patients were randomized into four groups (titanium healthy, titanium mucositis, zirconia healthy, and zirconia mucositis) in a follow-up RCT comparing soft tissue surrounding zirconia and titanium implants at clinical, microbial, and histological levels. After three months, buccal soft tissue biopsies were taken to evaluate the number of inflammatory cells and the distance of the junctional epithelium, with half of the patients instructed to continue performing oral hygiene measures and the other half to skip it around the implants for 3 weeks to induce experimental mucositis. Except for the junctional epithelium in the mucositis groups being longer than in the healthy groups, there were no other visible variations between the groups. In the location of the elongation, this was an overall rise in epithelial height due to edema in the mucositis groups. In summary, in healthy circumstances, both implants produced results that were comparable. In experimental mucositis conditions, zirconia implants showed significantly lower plaque and bleeding scores [17].

In a further study, the gingival biopsy was performed near zirconium and titanium oxide healing covers after six months in patients who received dental implants to measure microvascular densities and inflammatory infiltrates in a semi-quantitative way. The results showed a higher level of the inflammatory infiltrate (plasma cells, lymphocytes, and histiocytes) and a significant increase in the microvascular density within the submucosa in the titanium samples than in the zirconium oxide samples [18], pointing to the possibly higher biocompatibility and suitability of zirconia with a lower potential for bacterial colonization [19].

An RCT was conducted to investigate if treating titanium abutments with the plasma of argon and steam could enhance soft tissue adhesion and sealing abilities. The number of chronic inflammatory cells and the collagen band of the soft tissue around titanium abutments after seven days of the second surgery were evaluated. The results showed no significant differences regarding the probability of healing and inflammation between the plasma, steam, and control groups [20]. The effect of different surface treatments on the healing of soft tissue surrounding hydrophobic machined, chemically modified hydrophilic acid etched titanium or modified hydrophilic acid etched titanium–zirconium alloys was investigated after eight weeks from their placement. The highest levels of epithelium and sub-epithelial connective tissue contact were detected in zirconia-treated abutments with no statistically significant difference. The internal sub-epithelial connective tissue zone displayed a collagen fiber alignment perpendicular to both chemically modified hydrophilic acid etched titanium and zirconia abutments. In contrast, bundles of connective tissue fibers arranged in a parallel way were seen in the hydrophobic machined titanium. These findings suggested that zirconia abutments may be able to improve the soft tissue adherence of titanium implants at the transmucosal level due to their hydrophilic properties at the nanostructure level, their high wettability, and their enhanced free energy [21].

In another RCT, soft tissue was harvested for histological analysis three months after implant placement in the mandibles of edentulous patients. The implants were immediately loaded with investigational abutment materials (titanium, zirconia, zirconia covered with feldspar ceramics, and poly ether ether ketone (PEEK)). There were no appreciable variations found in the quality of collagen fibers or the presence of rete pegs in any of the abutment materials, although zirconia concealed with feldspar ceramics was marginally more effective than titanium in decreasing chronic inflammatory cells such as neutrophils and mononuclear cells [22].

**Figure 2:**
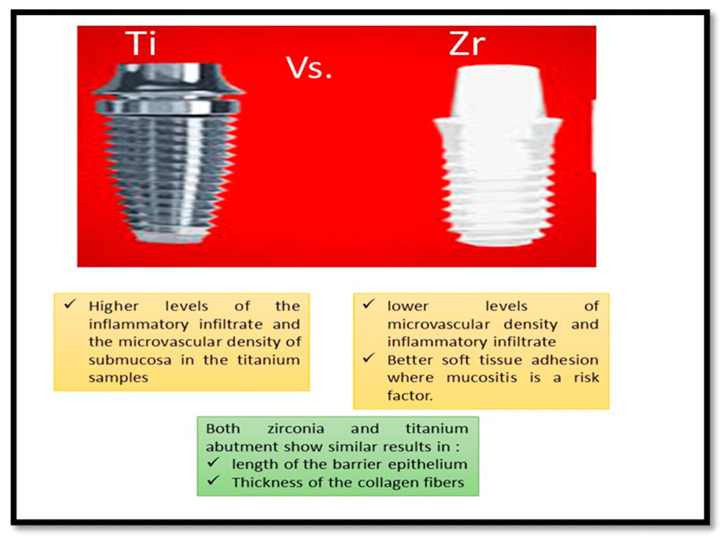
Comparison between zirconia and titanium abutments for dental implants, revealing their histological effects on soft tissue health.

Accordingly, the histological analyses conducted in several studies demonstrated that zirconia and titanium abutments function similarly in preserving the health of the soft tissue surrounding dental implants. In healthy settings, both materials showed comparable inflammatory reactions and attachment traits. However, Zirconia showed benefits in several situations, especially in experimental mucositis, where it led to a lower score with respect to bleeding and plaque accumulation. Zirconia proved to be more biocompatible than titanium, as seen via the lower microvascular density and inflammatory infiltrate levels (Figure 2).

These controversial findings, in relation to the systematic reviews investigating titanium and zirconia implants’ survival rates [12,13], could be attributed to the short duration time, as most tissues were harvested within a period ranging from 7 days to 6 months for histological assessment in the studies included in this section.

### 3.2. Animal Studies

Six beagle dogs had titanium and zirconia implant abutments inserted into their mandibles. The animals were sacrificed nine months following implant/abutment implantation to undergo histopathological examination. The findings revealed no appreciable variations in biological breadth, barrier epithelium length, the proportion of collagen fiber thickness, and connective tissue height. Still, the proportion of blood vessels in the connective tissue was higher around titanium in comparison to the zirconia abutments. This was referred to as less plaque accumulation around the ceramic abutments, which leads to a decrease in the inflammatory reaction with lower neo-vascularization to transport inflammatory cells, oxygen, and nutrients to the tissues [23]. Six Labrador dog mandibles were used in another study to investigate the soft tissue barrier to various implant abutments, including titanium, zirconia, and custom-built gold-colored alloys (Au-pt). Following the extraction of the maxillary premolars, as well as all mandibular premolars, four implants were positioned, and healing abutments were linked. The healing abutments were taken out after a month, and two titanium, zirconia, and Au-pt abutments were positioned in random order. No discernible variations were seen between zirconia and titanium abutments five months following the initial abutment connection surgery with respect to the percentage of blood vessels, collagen fibers, and biological width dimension. However, compared to titanium, the percentage of leukocytes in the barrier epithelium at zirconia abutments was lower, with no significant differences. The lower leukocyte percentage suggests that zirconia offered favorable circumstances for epithelial adhesion in the creation of an appropriate peri-implant mucosal seal [24].

In a dog model, the early soft tissue reaction (28 days) after placement of zirconium and titanium healing abutments was assessed in both inflammatory and healthy environments. The abutments were distributed into four groups: zirconia or titanium abutments that were either ligated or non-ligated. The ligation approach was employed to generate peri-implant mucositis. Tumor necrosis factor (TNF)-α and interleukin (IL)-1β immunohistochemical analysis showed the increased expression of positive inflammatory cells in soft tissues next to ligated zirconium and titanium healing abutments compared to non-ligated groups [25].

A histological study was further performed on tissues around implants loaded with abutments of different materials, such as one-piece titanium, two-piece titanium, zirconia, and lithium disilicate in the mandible and maxilla of minipigs after the extraction of their premolars and molars. The soft tissue biopsy taken after 6 months for determining the length of the sulcular depth, junctional epithelium, connective tissue, biological width, and first cervical bone–implant contact (distance between implant shoulder and first cervical bone–implant contact) showed no significant difference between different abutments, with an exception for the length of the junctional epithelium, which was significantly lower in zirconia as compared to one-piece titanium [26].

To further assess soft tissue integration, modified zirconia abutments (self-glazed with nanotopography) were implanted into rabbit models in the mandibular anterior region for four weeks. These abutments were compared to titanium and normal polished zirconia. The adjusted nano-scale zirconia surface greatly enhanced soft tissue attachment, as demonstrated via histological analysis. Better collagen fiber organization and closer contact between the soft tissue and the zirconia surface via hemidesmosomes indicated that the nano-topographical surface stimulated the formation of these structures, resulting in a more powerful and durable bond [27].

As a conclusion for the histological evaluation in animal studies, both zirconia and titanium performed similarly in terms of biological breadth, the length of the barrier’s epithelium, and the thickness of collagen fibers (Figure 2).

## 4. Immunohistochemical Assessment

### 4.1. Peri-Implant Soft Tissue Inflammatory Response

Immunohistochemical (IHC) analysis of inflammatory cells in mucosa surrounding the implant is a key factor in assessing the pathogenesis of soft tissue inflammation. A greater number of inflammatory cells were discovered around failing dental implants depending on the location and type of abutment material [28]. The cellular and vascular densities of peri-implant mucosa around titanium abutments were analyzed at different healing times. Soft tissues were retrieved after two, four, six, eight, and twelve weeks and stained with CD3 (T cells), CD20 (B cells), CD68 (macrophages), and CD34 antibodies. Between four and eight weeks of healing, B- and T-cells appeared with a reduction in cell density. Neutrophils were limited to the part of the tissue that is adjacent to the junctional epithelium, whereas macrophages were uniformly dispersed throughout the connective tissue along the abutment/tissue interface. After two to eight weeks of recovery, the density of the vascular structures decreased [29]. Inflammation triggered by titanium abutments is most probably mediated by B-cells, as by comparing the soft tissues’ responses around PEEK abutments and titanium using leukocyte common antigens (LCAs) and CD3, CD20, and CD68 antibodies, the number of LCA+ and CD 68+ cells was significantly lower in the titanium group, while CD 20+ and CD3+ were found in an expressively greater amount [30].

Regarding samples of human patients, soft tissue inflammation surrounding titanium and zirconia abutments was examined by evaluating the levels of LCAs, nitric oxide synthase (NOS1 and NOS3), CD20, vascular endothelial growth factor (VEGF), and CD3. In contrast to zirconia, the titanium group showed significantly higher expression with respect to the previous markers; this could be due to the increased number of bacteria that were stuck to the titanium samples and caused a more severe inflammatory response [18]. The increased expression of inflammatory mediators could promote angiogenesis, which subsequently influences the severity of inflammation [31].

Another human study revealed more CD3+ cells around zirconia abutments compared to the titanium group, which may reflect a Th2 response and the production of anti-inflammatory cytokines. On the contrary, more CD20+ cells were found around the titanium group [32]. Matrix metalloproteinase (MMP)-2, 3, 8, 9, and 13 expressions in peri-implant soft tissue around zirconium oxide and titanium abutments were further investigated in human subjects. After six months, MMP-8 and 9 were found to be significantly higher around titanium samples, while MMP-2, 3, and 13 showed no notable distinctions between the two groups. The authors deduced higher rates of soft tissue healing around titanium abutments than that of zirconia [33] due to higher rates of MMP-8 and 9 involved in the healing mechanism of periodontitis [34,35].

In a human histological study, zirconia abutments exhibited a statistically significant difference in CD20+ cells compared to titanium abutments. A well-regulated local immune system is indicated by the presence of CD3+ T cells in the soft tissue around implants. However, a high concentration of CD20+ cells may be a marker of sensitization and the maturation of lymphocytes into plasma cells [36].

Implant failure could arise from the immune cells’ modulation via titanium particles released from the implant’s surface. In a human study, peri-implant gingival tissues were taken from patients who had successful implants, failed implants, and no implants at all. Using the results of an IHC analysis, an immunoreactive score was created to assess the expression of CD68 and CD3. IHC staining verified the presence of immune cells and titanium particles in the tissue samples, with greater lymphocyte and macrophage infiltration in the implant failure samples. In instances of unsuccessful implants, there was a greater degree of positive expression of CD68 and CD3 compared to successful implants. Furthermore, the gingival tissues obtained from individuals without dental implants exhibited the same markers’ negative expression. These results demonstrated that titanium particles influence the polarization of macrophages and lymphocytes in the gingival tissues around implants [37].

### 4.2. Peri-Implant Cell Adhesion Response

Immunohistochemical analyses of the peri-titanium implant extracellular matrix of healthy tissue were found to be very comparable to that of the gingival tissues regarding the patterns of fibronectin (FN); laminin; and collagen type I, III, IV, and VII distribution. Still, the peri-implant connective tissue contains a larger amount of collagen type V and VI, which may function as a mechanical barrier to bacterial invasion [38]. The dissemination of desmoplakins and keratins in the human gingiva and peri-implant mucosa around titanium abutments that had been placed for two years was compared in an earlier study. The peri-implant mucosa stained much less for desmoplakins (I and II) than gingiva according to the results. Additionally, variations in keratin (13 and 19) staining were observed, suggesting a distinct phenotype in the functional epithelium around the titanium abutment. The functional characteristics of the peri-implant junctional epithelium may be linked to the co-expression of keratins (13 and 19), or it may signal a disruption in the normal keratin production process surrounding dental implants [39].

In a rat model, the peri-implant epithelium (PIE) seal after four weeks around titanium and zirconia implants was investigated via an IHC assessment of Laminin-332 expression with light and electron microscopes. Laminin-332 manifestation on the zirconia implant showed a comparable pattern to that of titanium at the light microscopic level. However, electron microscopic investigations revealed that the zirconia surface showed significantly less sticky structures and appeared to establish a poor epithelial barrier at the tissue–implant contact, while the epithelial tissue surrounding titanium showed a structure similar to that of a natural tooth. Furthermore, the in vitro cell adhesion of oral epithelial cells to zirconia plates was significantly weaker compared to the titanium plates, while the number of migrating oral epithelial cells on the zirconia plates was significantly greater than on titanium plates [40].

In rat oral epithelial cells, the localization of integrin β4 subunit (Inβ4) surrounding titanium and zirconia abutments was further studied. Following four weeks of implant insertion, PIE morphology and Inβ4 localization around both groups revealed comparable outcomes. Accordingly, it could be inferred that the sealing capability around titanium and zirconia was similar. Nevertheless, both showed noticeably shorter Inβ4-positive bands than those found in the JE surrounding normal teeth [41].

In a rat model, soft tissue adhesion in response to titanium and zirconia abutments was immunohistochemically evaluated against laminin-5, which is a typical structure in the internal basal lamina. Robust immunoreactivity was detected at the interface between both titanium and zirconia with the adjacent epithelium, indicating that both materials displayed excellent cell adhesion properties [42]. Little variations have been noted in the peri-implant mucosa surrounding both groups in a meta-analysis. Both materials showed a typical supracrestal tissue attachment. Zirconia implants exhibited a comparable pattern of connective tissue adhesion transgingival to titanium implants. Laminin-5 expression was detected via immunohistochemical analysis at the interface of the two groups, suggesting active epithelial tissue attachment [43].

To elucidate the impact of titanium implant surface modification on wound healing and cellular attachment for biological sealing in soft tissue around the implant, hydrophilization relative to a titanium disc was assessed. Samples were taken 3, 7, and 14 days after treating titanium implants with plasma and carrying out insertions into a rat’s upper molar socket. The findings revealed that, in comparison to the control, the peri-implant connective tissue in the plasma-treated group was wider, and epithelial downgrowth was suppressed. Furthermore, integrin-α2, α5, and β1 strong immunoreactions were seen in the implant contact area of the plasma-treated group compared to the control [44].

Overall, IHC analyses of ECM proteins around titanium and zirconium implants demonstrated great upregulation in important cell adhesion proteins such as fibronectin, collagen, integrins, and laminin in both titanium and zirconium implants, which is essential for effective tissue integration. The strong expression of the cell adhesion protein is present in titanium implants, which promotes both osseointegration and soft tissue integration.

A summary of the histological and immunohistochemical studies of peri-implant soft tissue is displayed in Table 1.

### 4.3. Effect of Abutment Surface Characteristics on Peri-Implant Soft Tissue Response

Features of the abutment surface could play a crucial part in the soft tissue reaction around the implant. Researchers have found a spatial relationship between the occurrence of bacterial colonization and the roughness of intraoral materials. This relationship suggests that the trans-mucosal portion of the abutment’s surface characteristics may contribute to bacterial population reduction, thereby reducing plaque formation and the ensuing peri-implant soft tissue inflammation [54,55]. Yet, there is no perfect implant surface that decreases bacterial adhesion and increases soft tissue adherence [56].

Fibroblasts are the prime cells of gingival connective tissues, being essential in preserving the soft tissue seal at the transmucosal implant area and controlling its integrity [57]. Following implant insertion, they are the initial cells that replenish the injury and release collagen in the extracellular matrix, which initiates the renewal of soft tissue around the implant [2]. Among the primary variables influencing soft tissue adhesion at the surface of the implant are surface energy, topography, and therapeutic approaches [58]. Increasing the surface hydrophilicity or, in other words, the wettability of the material surface would dramatically increase the cell’s attachment [59,60].

Human fibroblasts and epithelial cells’ proliferation activity and attachment behavior were examined on various surface topographies of zirconia and titanium abutments (machined, smooth, and rough). When compared to titanium, fibroblasts proliferated at noticeably higher rates on various zirconia surface topographies. On polished and machined surfaces, epithelial cells attached to titanium alloy expressed more vinculin, the focal adhesion protein, than those adhered to zirconia. Conversely, a rough surface made fibroblasts’ cellular adherence easier while making epithelial cells’ cellular adhesion harder [46]. Several topographic modifications to dental implants’ transmucosal sections can significantly affect fibroblast bioactivities, especially cell adhesion. An in vitro study proved that increasing titanium implant surfaces’ hydrophilicity using super-hydrophilic titanium oxide nanotubes significantly upregulated the appearance of cell adhesion molecules in human gingival fibroblasts, such as integrin α2, focal adhesion kinase, FN1, and collagen type 1, indicating a fast, tight, and strong connective tissue seal around the implant transmucosal portion [61].

Previous investigations state that the epithelium at the peri-implant area exhibits a weak adhesion to titanium abutments via laminin [62], failing to create a tight soft tissue barrier around implants. The tooth is attached to the junctional epithelium by means of hemidesmosomes that are created by integrins β4 in the internal basal lamina and laminin γ^2^. Peri-implant epithelium binds with titanium abutments by hemidesmosomes, which realize the localization of laminin γ^2^ [63,64]. This was not the case with zirconia abutments [65]. Accordingly, surface modifications for zirconia were necessary to enhance the biological seal, with marked effects on cellular behavior and protein adsorption simultaneously [66,67]. In this context, integrins are vital transmembrane receptors responsible for cell–matrix interactions and cell–cell adhesion [68]. The expression of integrins can give a good impression about the reliability of peri-implant soft tissue seals. Different additive manufacturing preparations (fully rough surface, upper machined + lower rough, rough upper surface + lower machined, and fully machined) for titanium abutments were made to investigate their effect on cell adhesion. Forty samples of peri-implant mucosa were collected after 30 days of implant placement. Fully rough abutment surfaces had the greatest effect on modulating the cellular behavior of epithelium, and this is demonstrated by a significant increase in the integrin β1 subunits favoring epithelial cell migration and strengthening junctional epithelium attachment around implant surfaces. On the contrary, the surface topography of fully machined smooth surfaces, e.g., the upper and lower machined surfaces, revealed no noteworthy increase in the integrin levels [62].

Investigating the subepithelial connective tissue attachment to modified and conventional sandblasted, large grit, and acid-etched (SLA) titanium abutments was conducted in a dog model using monoclonal antibodies relative to FN and proliferating cell nuclear antigens (PCNAs). The initial signs of positive FN and PCNA staining were observed near both implant surfaces on day four. The levels of FN and PCNA antigen reactivity increased over time in both groups, while PCNA appeared to be more pronounced upon modified SLA implantation [49]. Soft tissue reactions surrounding machined and acid-etched titanium healing caps were further evaluated for inflammatory cell infiltrate B and T lymphocytes, microvascular density, NOS 1 and 3, histiocyte, VEGF, and the proliferative activity of Ki-67. All markers showed higher expression in the acid-etched group, suggesting a higher rate of restoration correlated to an increase in inflammatory processes [45].

Applying zinc oxide nanocrystals on zirconia abutments with the aim of achieving a proper early soft tissue seal encourages the progress and adhesion of epithelial cells on zirconia surfaces through the upregulation of laminin 332 and integrin β4 expression via the controlling PI3K/AKT pathway. PI3K might inhibit apoptosis and enhance oral epithelial cell multiplication and attachment. The binding of integrin β4 and laminin 332 could form a matrix for the basement membrane and allow strong epithelial cell attachment with the basement membrane by connecting the cytoplasmic site with the intracellular cytoplasmic plaque. Following its binding, the activation of PI3K via phosphorylation takes place [69].

An enamel matrix derivative (EMD) was applied via two different doses, 25 ug/mL and 100 ug/mL, on zirconia discs. Type I collagen expression was stimulated by a high concentration (100 ug/mL) of EMD, which promotes fibroblast attachment; in addition, this high dose boosted the expression of transforming growth factor β1, which is a chief growth factor governing different cell production and integrin expressions. Fibronectin increased with 100 ug/mL of EMD as well. Yet, further investigations to determine the utmost concentration of EMD for the optimal effect on soft tissue attachment are needed [70].

An excimer laser used for photo-functionalization was employed recently to increase the bond between fibroblasts and implant abutment surfaces [2,4]. In two groups of zirconia discs, with one control group receiving no laser excitation and an experimental group receiving excimer laser excitation, a significant increase in the expression of two cell attachment proteins (collagen type Iα and integrin β1) in the investigated groups at 6 and 24 h intervals was notable, suggesting strong adhesion between zirconia and fibroblasts [71]. Surface modifications using ultraviolet light and cold plasma for zirconia disc surfaces were utilized to enhance the implant wettability and cellular attachment of human oral keratinocytes. Quantitative RT-PCR results showed a three-times higher expression for laminin γ^2^ in the plasma group than in the ultraviolet or control group. The same results were obtained with integrin β4 expression. These high expression levels demonstrate an enhanced initial attachment capability and the spreading of cells on plasma-treated specimens [72]. Additional in vitro studies demonstrated that the application of hydrides to titanium/zirconia surfaces boosted the production of metallopeptidase inhibitor-1 (TIMP1) and reduced the MMP1/TIMP1mRNA ratio, which accounts for the high concentrations of native fibrillar collagens found in the extracellular matrix [73].

An experimental in vitro investigation introduced laser-lok, which is a laser-modified titanium disc surface with identical microgrooves, and its effect was tested on human gingival fibroblasts related to unmodified titanium and zirconia discs. The findings revealed a substantial difference in the expression of TNF-α and IL-10 between the titanium and Laser-lok groups but not between those groups and the zirconia group. Conversely, the zirconia group had the highest levels of FN and integrin gene expression, followed by the Laser-lok group and the titanium group [58].

A new in vitro organotypic three-dimensional model of reconstructed human gingiva on a lamina propria (a collagen hydrogel filled by fibroblasts) was constructed. The expressions of keratin 4, 19, Ki67, laminin 5, and collagen IV were evaluated on two different titanium abutment surfaces: one that had been anodized and the other that had not been. There was no discernible variation in the epithelial attachment on either surface. The basal layer contained proliferating Ki67-positive keratinocytes, and the hydrogel–epithelium interface showed the positive expression of collagen IV and laminin 5. While junctional epithelial keratin 19 was only found in the undifferentiated basal cell layers, keratin 4 was detected in the higher epithelial cell layers [48].

Abutment materials could further strengthen soft tissue attachments in ways other than those focusing on fibroblasts and epithelial cells. This new route functions by approaching the immune microenvironment surrounding the implant. In this context, studies were conducted to investigate the impact of various nanostructured zirconia on macrophage morphologies and the subsequent influence on gingival fibroblast activity. Three distinct nanostructured zirconia disc surfaces comprised blank machined zirconia (BMZ), self-glazed zirconia (SGZ), and coated self-glazed zirconia (CSGZ), and they were used to cultivate the mouse-derived macrophage cell RAW264. After three days, a conditioned medium (CM) was taken from each group and administered to human gingival fibroblasts to replicate the in vivo milieu. Both inflammatory M1 and anti-inflammatory M2 macrophages had their macrophage polarity controlled by each of the several disc surface groups. However, BMZ produced a substantial amount of IL-10 and showed a greater tendency towards the M2 phenotype. Additionally, the gingival fibroblasts’ generation of the extracellular matrix and proliferation was enhanced by the macrophage CM collected from around the three nanostructured zirconia materials, particularly when combined with BMZ followed by CSGZ. The fibroblasts also expressed more collagen I, vinculin, and fibronectin. Thus, macrophages were prone to favor more adhesion on BMZ and CSGZ surfaces [74].

To summarize, surface modifications are advantageous for both zirconium and titanium abutments in enhancing the soft tissue response surrounding the implant. Surface roughness enhances soft tissue cell attachment by improving fibroblast and epithelial cell adhesion. A stable oxide layer that is formed through oxidation or anodization improves biocompatibility and promotes improved tissue integration. Plasma spraying enhances the biological response through osseointegration and soft tissue adhesion by imitating the structure of genuine bone. Polishing creates a smoother surface and lowers bacterial adherence, thus helping to improve the soft tissue response. Silica coatings can increase the bioactivity of the surface, facilitating better soft tissue integration and lowering inflammatory responses.

Upon surface modification, titanium abutments exhibited improved adherence of soft tissue, decreased the buildup of bacteria, and decreased risks of inflammation. Regarding zirconium abutments, they showed better soft tissue integration with less inflammation and bacterial adherence when their surfaces were polished and roughened.

A bibliometric analysis of the 50 most-cited articles in soft tissue integration assessed the scientific advancements in this area. The surface structure of dental implant materials is crucial for the integration of soft tissue with dental implants. The integration of soft tissues has captured attention in recent years; however, numerous experiments are still required to enhance the compatibility of soft tissues with new materials. It was noted that among the 50 most frequently cited articles, titanium emerged as the predominant implant material utilized in both animal studies and clinical trials. The most common surface treatment involved changing surface roughness, while research on creating new coatings was limited. The majority of the coatings were metal compound coatings of titanium and zirconium [75]. The molecular analysis of gene expression in human gingival fibroblasts/epithelial cells in soft tissues related to zirconia and titanium abutments are revealed in Table 2.

## 5. Proteins Expression in Peri-Implant Crevicular Fluid

**Figure 3:**
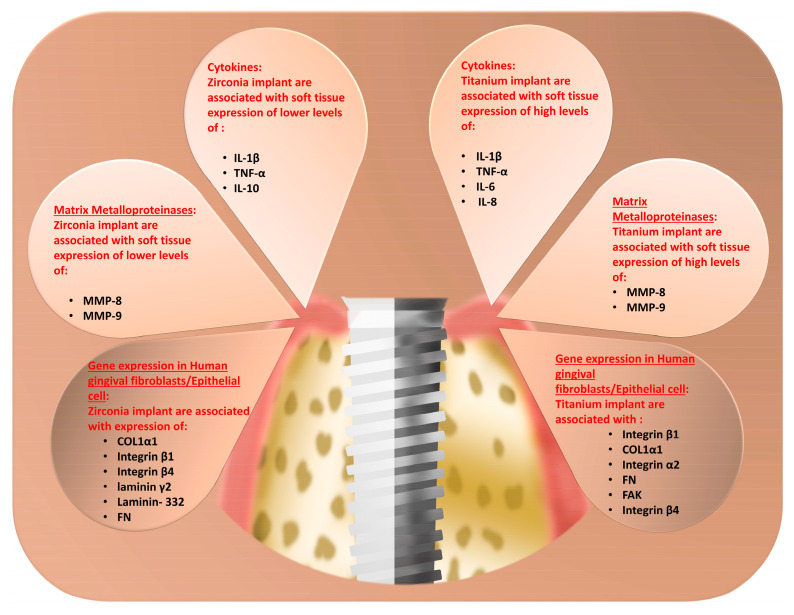
Differential molecular profile analysis between zirconia and titanium dental implants, illustrating distinctive patterns in pro-inflammatory cytokine expression, matrix metalloproteinase activity, and gingival fibroblast/epithelial cell-specific gene expression patterns at the implant–tissue interface.

Studying the cellular and metabolic processes surrounding dental implants and abutments can be carried out effectively using bimolecular analyses of the expressed proteins in peri-implant crevice fluid (PCF) (Figure 3) [76]. In a prospective clinical trial conducted to compare the levels of interleukin IL1β and IL6 in GCF via an enzyme-linked immunosorbent assay (ELISA) with respect to titanium and zirconia abutments after four months of prosthetic function, a significant increase was observed with respect to IL6 in the GCF around the titanium abutments, with no significant difference observed for IL1β expression. Furthermore, an analysis of the mean concentrations between IL6 and IL1β revealed that IL6 was significantly present in higher amounts near titanium and ceramic abutments than IL1β [47].

A prospective clinical study was further carried out to assess MMP-8 levels in PCF in 12 partially edentulous patients with right and left randomization. After the abutments were inserted, pocket probing depths and PCF sampling were assessed 1, 3, and 12 months later. Titanium abutments revealed significantly greater levels of MMP-8 and probing depths than the abutments of zirconia within 1 and 3 months, but no notable variations were found in the 12 months between them [50]. After 22 months of clinical loading, a second cross-sectional study using a fluorescent bead-based immunoassay instrument was carried out to evaluate variations in the protein expression of the pro-inflammatory cytokines and bone metabolism mediator in the PCF next to titanium and zirconia trans-mucosal abutments in 46 participants with identical implant systems. No significant differences between titanium and zirconia abutments were notable regarding pro-inflammatory cytokines such as IL2, IL4, IL7, IL8, IL12, IL 10, IL13, and TNF-α, while leptin showed a significant increase around titanium rather than zirconia abutments [51].

In a further cross-sectional trial, one zirconia implant with one contralateral natural tooth and a previously placed titanium implant if present were assessed after a minimum of one year of function in 36 systemically healthy subjects. The levels of IL1β, IL1 receptor antagonists (RAs), IL6, IL8, IL17, fibroblast growth factor (b-FGF), granulocyte colony-stimulating factor (G-CSF), granulocyte–monocyte colony-stimulating factor (GM-CSF), interferon, macrophage inflammatory protein (MIP-1β), and TNF-α and VEGF were evaluated in PCF/GCF. Additionally, the following clinical parameters were evaluated at six sites surrounding each implant or tooth: bleeding on probing (BOP), probing depth (PD), gingival index (GI), and plaque index (PI). When comparing zirconia implants to teeth, the mean PI was much lower, while the mean GI, PD, and BOP were notably greater. The expression of IL1RA, IL8, G-CSF, MIP-1β, and TNF-α at zirconia implants and teeth was found to be correlated. There were significant increases in IL1β and TNF-α at zirconia implants compared to tooth, yet no significant differences were found between zirconia and titanium implants. The levels of GM-CSF, MIP-1β, IL1RA, and IL8 at zirconia and titanium implants were found to be correlated. According to the scientists’ conclusions, correlations between the expression of several biomarkers at zirconia and titanium implants, as well as teeth and zirconia implants, may represent patients’ unique inflammatory response patterns that were not locally altered by the implant material [52].

In an RCT conducted to assess the inflammatory impact of singular abutments bonded onto titanium bases on tissues surrounding the dental implant, the levels of IL-1β via ELISA were measured from the GCF of both groups; the titanium test abutments made via computer-aided design/computer-aided manufacturing (CAD/CAM) were bonded onto titanium abutments, while the control abutments were made up of only one-piece CAD/CAM titanium abutments. PCF was obtained 6 and 12 months after surgery. The levels of IL1β in both groups were higher than the borderline. However, there was no discernible difference between singular abutments bonded onto titanium bases and singular abutments in terms of their effect on the inflammatory state of the surrounding tissues of dental implants [53].

In summary, increased levels of pro-inflammatory cytokines (TNF-α and IL-1β) and MMPs in the gingival tissue around titanium implants were demonstrated more than zirconium implants. Shortly (three to four months after implant insertion) and in the long term (twelve months), no significant difference in the pro-inflammatory cytokines was detected. This early inflammatory response could be an indication of early inflammatory processes required for tissue turnover that subsided after that.

## 6. Bacterial Colonization

In a clinical study comparing early bacterial colonization to zirconia and titanium abutment surfaces, sulcular plaque samples were obtained at two weeks and three months post-operatively. The detection and counting of the number of bacteria was performed using 16S rDNA. Findings exhibited that zirconia and titanium abutments contained similar numbers of *Aggregatibacter actinomycetemcomitans (A. actinomycetemcomitans)*, *Porphyromonas gingivalis (P. gingivalis), Prevotella intermedia, Tannerella forsythia (T. forsythia), Peptostreptococcus micros, Fusobacterium nucleatum (F. nucleatum)*, and *Treponema denticola (T. denticola)* at both studied intervals with no significant differences [77]. Still, other studies revealed that zirconia discs harbored less bacteria [78,79].

Another study was conducted to identify and quantify species identities related to either titanium or zirconia implant abutments. Zirconia abutments were positioned in the anterior region of the maxilla, whereas titanium abutments were positioned in the posterior area of the maxilla and mandible. Microbiological samples were harvested at baseline–implant loading three months and six months after the intervention, and they were analyzed using DNA-Checkerboard and 16S-rDNA pyrosequencing. Species that were common to all sites belonged to the genera *Fusobacterium, Prevotella*, *Actinomyces*, *Porphyromonas*, *Veillonella*, and *Streptococcus (S)*. Titanium-related sites presented the highest total microbial count and high counts of pathogenic species [80]. In a similar study evaluating the microbial colonization on titanium or zirconia abutments along three years of function, *A. actinomycetemcomitans* serotype A and *F. nucleatum* were discovered in greater numbers in the zirconia and titanium abutment peri-implant biofilms. It was discovered that the two materials’ microbiological profiles and bacterial numbers varied over time. During the first 12 months, subgingival samples from titanium-related areas showed the greatest values of genome numbers. Conversely, in the next two years, zirconia-related sites showed the greatest numbers and were more vulnerable to the presence of microorganisms three years after loading [81].

The evaluation and comparison of bacterial adherence on the surfaces of zirconia and titanium abutments were performed in a randomized clinical experiment. Both the anterior and posterior regions of the mouth cavity received abutments. After being exposed to the oral cavity for 24 h, the investigated materials displayed bacterial colonization, and suspected periodontal pathogens such as *T. denticola*, *T. forsythia*, and *P. gingivalis* were isolated from various substrates using DNA chequerboard analysis. Across all categories, *Streptococcus* spp. were the most frequently prevalent species. Overall, the group using zirconia abutments displayed reduced bacterial levels. Bacterial adhesion did not significantly alter depending on whether the abutments were placed in the anterior or posterior region [82].

Moreover, by comparing the percentage of surface covered by bacteria on commercially pure titanium and zirconium oxide disks using scanning electron microscopy, the zirconium oxide surfaces have a significantly reduced presence of bacteria [19]. This discovery may have implications related to zirconium oxide’s electric conductivity. Depending on the substratum’s specific resistivity, electron transfers may contribute to bacterial adhesion to surfaces. More firmly, adherent bacteria were those that contributed electrons to the substratum rather than those that took electrons from it [83].

A further clinical study was carried out to determine the microbiota composition in the biofilm after one, three, and six months of loading with respect to either titanium or zirconia abutments. A 16S rDNA gene-based metagenome approach identified a dominant pattern of bacteria from five phyla, namely, *Firmicutes, Fusobacteria*, *Actinobacteria*, *Bacteroidetes*, and *Proteobacteria.* Of those identified, *Firmicutes* showed a higher prevalence (over 45%) for both titanium and zirconia groups, with the prevalence of other bacterial phyla being similar in both groups over time [84].

To assess the relationship between the topography and surface roughness of the materials and the biofilm production on zirconia or titanium substrates, another clinical trial was carried out. After the specimens were exposed to the mouth for 24 h, there were no variations in the total biofilm development indexes when stained with 1% neutral red. Furthermore, there was no discernible relationship between the means of the surface’s roughness and the overall amount of biofilm that was formed. The zirconia group demonstrated the greatest means of surface roughness and the lowest microbiological count compared to titanium. Moreover, the disc implantation region (posterior or anterior) had no effect on the development of biofilms [85].

The number and quality of bacterial composition of early and mature biofilms were evaluated across implant/abutment materials with varying surface roughness values (Ra). Using RT-qPCR, the total count of bacteria was determined. Using microarrays, the presence and growth of 20 chosen bacterial species were evaluated following the implantation of mandibular acrylic devices containing four disks (titanium abutment, zirconium dioxide abutment, machined titanium, and sand-blasted acid-etched titanium) after 3 days and 31 days to evaluate early and mature biofilm formation.

The results showed that the isolated microflora included suspected periodontal pathogens of the orange complex, including *Parvimonas micra*, and species linked to health, like the *S mitis* group. The total number of bacterial cells in the biofilms generated on zirconia was larger than that of biofilms formed on titanium surfaces with significantly lower roughness values. These discrepancies can be attributed mostly to the various surface roughness values [86]. To determine the primary determinant for bacterial adhesion for each material, in vitro research of bacterial adhesion on various surfaces of titanium and zirconia implants was conducted. The results were correlated with surface roughness and surface hydrophobicity. After being treated with bacterial suspensions of *S. sanguinis* and *Staphylococcus epidermidis*, zirconia and titanium specimens with varying surface textures and wettability were assessed using the fluorescent dye CytoX-Violet and an automated multi-detection reader. Surface roughness (Ra) variations did not influence the adhesion of *Staphylococcus epidermidis* in any way. On the other hand, *S. sanguinis* adhesion increased with increasing Ra. In contrast, *Staphylococcus epidermidis* but not *S. sanguinis* showed increased bacterial adherence on hydrophobic surfaces relative to hydrophilic surfaces. Compared to titanium surfaces, zirconia surfaces had a noticeably increased capacity for adhesion to *S. sanguinis*. However, no similar inclination was discovered for *Staphylococcus epidermidis*. The findings indicated that there was no difference in the capacity for bacterial colonization between titanium and zirconia implant material, and they also suggested that the bacterial species itself determines the primary adhesion factor. Moreover, the impact of implant surface wettability, surface texture, and substratum material on microbial adherence varies between bacterial species and does not precisely follow universal guidelines [87].

Furthermore, in both saliva-coated and non-saliva-coated conditions, an in vitro investigation examined the adhesion activity of periodontopathic bacteria to zirconia in comparison to titanium. To rule out surface roughness as a factor, all specimens were produced at a roughness of less than 0.1 µm. The findings revealed a decrease in initial attachment in saliva-coated specimens, but no significant variations were found in the initial attachment times of *P. gingivalis*, *P. intermedia*, or *A. actinomycetemcomitans* at 1 h. Similarly, there were no appreciable variations in the periodontopathic bacteria’s 48 h colonization. These results suggest that the hydrophobic interaction between substrates and bacteria on hydrophobic surfaces increases bacterial adherence [88].

A surface oxide layer covers titanium, and this oxide has mechanical and physical properties that are more in line with ceramics than with metals. This process could account for the comparable protein-binding characteristics observed in titanium and zirconium oxide and the reason zirconia did not exhibit decreased bacterial adhesion in prior investigations [89].

Moreover, a study investigating the effects of various titanium- and zirconia-polishing protocols on the colonization of oral bacteria was performed. Titanium and zirconia discs were divided into five groups: unpolished and polished with Brownie only, Brownie plus Greenie, Brownie plus Greenie plus Supergreenie, and CeraMaster Coarse plus CeraMaster polishing tips. The samples were sterilized and immersed in unstimulated saliva and then incubated in a liquid suspension of *Streptococcus gordonii*. The number of attached bacteria was counted 48 h after the diluted suspensions were inoculated. The results showed that inadequate polishing roughens surfaces and promotes microbial adhesion to titanium and zirconia. Sequential polishing to the finest finish polishing tips minimizes bacterial adherence to abutment surfaces. Zirconia exhibited less bacterial adhesion than titanium [90].

The previous literature provides valuable information about the microbiological composition and adhesion characteristics of bacteria relative to titanium and zirconia abutments. When compared to titanium, zirconia surfaces generally showed lower bacterial counts and adhesion rates, indicating possible benefits for reducing peri-implant infections. Important discoveries showed that, over time, both materials showed comparable patterns of bacterial colonization, with *Firmicutes* predominating in both groups. Furthermore, it has been demonstrated that, depending on the species, surface roughness and hydrophobicity affect bacterial adhesion in distinct ways.

Bacterial colonization in peri-implant–biofilm interphase studies is revealed in Table 3.

The limitations of this study are evident with respect to the need for a systematic review and meta-analysis to support the data presented. Yet, the number of RCTs focusing on histological and biological tissue interactions related to titanium and zirconium implants is limited. The only era that had available RCTs and observational and cohort studies that addressed the same outcomes comprised the detection of cellular and inflammatory infiltrates surrounding zirconium and titanium implants. To strengthen the findings and provide a more comprehensive understanding, future systematic reviews are recommended. These reviews should aim to include a broader range of studies to overcome the current limitations and enhance the reliability of the conclusions drawn. Moreover, in most available studies concerned with the histological and biological interactions and comparisons between titanium and zirconium implants included in the current review, the tissues were harvested shortly after implant placement (three to four months); therefore, evidence for long-term tissue–implant interaction is needed.

## 7. Conclusions

This review provides valuable insights into the peri-implant soft tissue reaction to zirconium and titanium abutments. Both materials have demonstrated satisfactory integration with the neighboring tissues, with comparable soft tissue attachment and inflammatory responses observed in histological studies. While titanium has been the traditional choice for abutments, zirconia offers advantages such as biocompatibility and aesthetic appeal. The present data analysis precludes a definitive preference for zirconia or titanium in terms of follow-up duration, research methodologies, and implant location region. However, several in vitro and in vivo investigations have shown that zirconia implants are a practicable substitute for titanium, offering comparable soft tissue integration, better soft tissue response, and biocompatibility.

The correlations between histological and immunohistochemical behavior and the biological analysis of peri-implant soft tissues provide important information for identifying a proper therapeutic strategy to ensure the longevity of dental implant restorations. Further research is needed to explore surface treatments and optimize outcomes in dental implant rehabilitation. In general, long-term success in implant dentistry depends on a grasp of the histological and biological elements of peri-implant soft tissue reactions to several abutment materials.

## Figures and Tables

**Table 1:** Histological and immunohistochemical analysis of peri-implant soft tissue.

Study	Study Design	Intervention	Analysis	Marker	Results
Degidi et al., 2006 [18]	RCT	Ti and Zi	Immuno-histochemical	CD3, CD20, LCA, VEGF, NOS1, NOS 3	Higher intensity expression around Ti More than Zi
Wang et al., 2021 [25]	Animal	Ti and Zn abutments either with ligation (L) or without ligation (N)	ELISA	TNF-α and IL1β	-TNF-α was significantly higher Zn (L) than Zn (N) groups on day 28 -TNF-α level was significantly higher in Ti (L) group on day 28 than that on day 0 -No significant differences in the levels of IL-1β
Tomasi et al., 2016 [29]	RCT	Ti	Immuno-histochemical	CD3, CD20, CD68, CD34	Decreasing cell density from 4 to 8 weeks of healing
Ilinkovic et al., 2022 [30]	RCT	Ti and PEEK	Immuno-histochemical	CD3, CD20, CD68, LCA	CD3 and CD20 were higher around Ti CD68, LCA higher around PEEK
Bambini et al., 2013 [32]	RCT	Ti and Zi	Immuno-histochemical	CD3, CD20	-More CD3 around Zi -More CD20 around Ti
Degidi et al., 2013 [33]	RCT	Ti and Zi	Immuno-histochemical	MMP 2, 3, 8, 9, 13	-MMP 8, 9 were higher around Ti -MMP 2, 3, 13 showed no difference among both groups
Serichetaphongse et al., 2020 [36]	RCT	Ti and Zi	Immuno-histochemical	CD3, CD20, CD68, CD138	-CD3 was higher in Zi group -CD20, CD68, and CD138 showed no difference between the 2 groups
Kheder et al., 2023 [37]	Cross sectional study/Human	Ti abutments either with successful implants or unsuccessful implants	Immuno-histochemistry	CD68 and CD3	In instances of unsuccessful implants, there was a greater degree of the positive expression of CD68 and CD3
Carmichael et al., 1991 [39]	RCT	Ti	Immuno-histochemical	Keratin 13, 19 and Desmoplakin I, II	-Different expression of keratin 13, 19 around Ti than around gingiva -Less expression of desmoplakin I, II around Ti than gingiva
Atsuta et al., 2019 [40]	RCT	Ti and Zi	Immuno-histochemical	Laminin-332	High expression around Ti than Zr
Ayukawa et al., 2020 [41]	Animal study	Ti and Zi	Immuno-histochemical	IntegrinB4	Similar results between both groups
Furuhashi et al., 2021 [42]	Anim al study	Ti and Zr	Immuno-histochemical	Laminin-5	Similar results between both groups
Harada et al., 2024 [44]	Animal study	Ti abutments (plasma treated and Untreated surface)	Immuno-histochemistry	Integrins	integrin α2, α5, and β1 showed strong immunoreactions in PL group as compared to control group
Degidi et al., 2012 [45]	RCT	Ti (acid etched, machined)	Immuno-histochemical	CD3, CD20, Histiocyte, VEGF, NOS1, NOS 3, Ki-67	More expression around acid etched than machined group
Nothdurft et al., 2015 [46]	In vitro	Ti and Zi	Immuno-histochemical	HGF-1, HNEpC	-Fibroblast showed higher expression around Zi -Vinoculin expression of epithilal cells showed higher expression around Ti
Negahdari et al., 2017 [47]	Prospective clinical study	Ti and Zn abutments	ELISA	IL6 and IL1β	-IL6 higher in Ti. -No statistical significance in IL1β
Roffel et al., 2019 [48]	In vitro	Ti (anodized, unmodified surface)	Immuno-histochemical	Keratin 4, 19, Ki-67, Laminin-5 Collagen IV	Similar results between both groups
Schwarz et al., 2007 [49]	Animal study	Ti (modified, unmodified)	Immuno-histochemical	FN, PCNA	-PCNA showed more expression around modSLA -FN showed similar results around both groups
Kumar et al., 2017 [50]	Prospective clinical study/human	Ti and Zn abutments	ELISA	Metalloproteinases (MMP-8)	-Ti abutments showed higher MMP-8 levels abutments at 1 and 3 months -No significant differences were found at 12 months
Barwacz et al., 2015 [51]	Cross sectional study/human	Ti and Zn abutments	Fluorescent bead-based immunoassay	Proinflammatory cytokines as (IL2, IL4, IL7, IL8, IL12, IL 10, IL13, and TNF α) Leptin	-No significant differences in Pro-inflammatory cytokines -Leptin higher in Ti group
Cionca et al., 2016 [52]	Cross sectional study/human	Ti, Zn abutments and natural teeth	Bio-Plex 200 suspension array	IL1β, IL1RA, IL6, IL8, IL17, b-FGF, G-CSF, GM-CSF, IFN, MIP-1β, and TNF-α and VEGF	-IL1β and TNF-α were significantly higher in zirconia implants than in teeth -No significant differences were found between zirconia and titanium implants
Chandra et al., 2023 [53]	RCT	Ti (singular abutments bonded onto Ti bases and singular abutment)	ELISA	IL-1β	IL-1β showed no significant difference between singular abutments bonded onto Ti bases and singular abutments

**Table 2:** Molecular analysis of gene expression in human gingival fibroblasts/epithelial cells upon the surface modification of titanium and zirconium implants. (+: detected, blank: not detected).

Study	Abutment Modifications		Adhesion Gene Expression in HGF/Epithelial Cell											
	Titanium	Zirconia	COL1α1	Integrin β1	Integrin α2	FN	FAK	Integrin β4	Laminin γ^2^	Laminin-332	MMP-1	TIMP-1	TGF-β	Vinculin
Esfahanizad eh et al., 2016 [58]	Laser-lok			+	+	+		+						
Wang et al., 2021 [61]	Super-hydrophilic TNTs		+		+	+	+							
Roth et al., 2022 [62]	AM healing			+										
Hu et al., 2023 [69]		ZnO nanocrystals						+		+				
Kwon et al., 2014 [70]		EMD	+			+							+	
Akashi et al., 2022 [71]		Excimer Laser	+	+										
Kobune et al., 2014 [72]		O2 plasma						+	+					
Gómez-Florit et al., 2014 [73]	Hydride implementation										-	+		
Wu et al., 2021 [74]		BMZ/CSGZ/S GZ	+			+								+

**Table 3:** Bacterial colonization in peri-implant–biofilm interphase.

Study	Study Design	Follow Up	Bacteria
Van Brakel R. et al., 2011 [77]	RCT	2 weeks and 3 months	*Aggregatibacter actinomycetemcomitans, Porphyromonas gingivalis, Prevotella intermedia, Tannerella forsythia, Peptostreptococcus micros, Fusobacterium nucleatum*, and *Treponema denticola*
Do Nascimento. et al., 2016 [80]	RCT	Up to 6 months	*Fusobacterium, Prevotella, Actinomyces, Porphyromonas, Veillonella*, and *Streptococcus*
de Oliveira. et al., 2020 [81]	RCT	1, 2 and 3 years	*A. actinomycetemcomitans* serotype A and *F. nucleatum*
Nascimento. et al., 2014 [82]	RCT	24 h	*Streptococcus* spp. and *L. casei* as well as putative periodontal pathogens, such as *T. denticola*, *T. forsythia*, and *P. gingivalis*
de Freitas. et al., 2018 [84]	RCT	6 months	*Firmicutes, Fusobacteria, Actinobacteria, Bacteroidetes*, and *Proteobacteria*
Do Nascimento. et al., 2013 [85]	RCT	24 h	Initial biofilm bacterial components such as: Bacterial suspensions of *Streptococcus sanguinis* and *Staphylococcus epidermidis*
Herrmann. et al., 2020 [86]	RCT	3 days and 31 days	*Streptococcus mitis* group and *Parvimonas micra*
Wassmann. et al., 2017 [87]	In vitro	48 h	*Staphylococcus epidermidis* and *S. sanguinis*
Egawa. et al., 2013 [88]	In vitro	Up to 2 days	*P. gingivalis, P. intermedia*, and *Aggregatibacter actinomycetemcomitans*

## Data Availability

Not applicable.

## Abbreviations

Abbreviation	Full name
*A. actinomycetemcomitans*	*Aggregatibacter actinomycetemcomitans*
AM	Additive manufacturing
Au	Gold
BGS	Brownie plus Greenie plus Supergreenie
BMZ	Blank machined zirconia
BOB	Bleeding on probing
BPG	Brownie plus Greenie
BRO	Polished with brownie only
CAD/CAM	Computer-aided design/computer-aided manufacturing
CD	Cluster of differentiation
CER	CeraMaster coarse
CM	Conditioned medium
COL	Collagen
CSGZ	Coated self-glazed zirconia
ELISA	Enzyme-linked immunosorbent assay
EMD	Enamel matrix derivative
*F. nucleatum*	*Fusobacterium nucleatum*
FAK	Focal adhesion kinase
FGF	Fibroblast growth factor
FI	Failed implants
FN	Fibronectin
G-CSF	Granulocyte colony-stimulating factor
GI	Gingival index
GM-CSF	Granulocyte monocyte colony-stimulating factor
HGF	Human gingival fibroblast
HNEpC	Human nasal epithelial cells
IHC	Immunohistochemical
IL	Interleukin
In	Integrin protein
IRC	Immunoreactive score
Ki	Kiel protein
LCA	Leucocyte common antigen
Ln	Laminin protein
MIP	Macrophage inflammatory protein
MMP	Matrix metalloproteinase
NOS	Nitric Oxide synthase
*P. gingivalis*	*Porphyromonas gingivalis*
PCF	Peri-implant crevice fluid
PCNA	Proliferating cell nuclear antigen
PD	Probing depth
PEEK	Polyetheretherketone
PI	Plaque index
PI3K/AKT pathway	Phosphoinositide-3-kinase-protein kinaseB/Activating receptor tyrosine kinases
PICF	Peri-implant crevicular fluid
PIE	Peri-implant epithelium
Pt	Platinum
Ra	Surface roughness
RCT	Randomized control trial
SGZ	Self-glazed zirconia
SI	Successful implants
SLA	Sandblasted, large grit, and acid-etched
*T. denticola*	*Treponema denticola*
*T. forsythia*	*Tannerella forsythia*
TGF	Transforming growth factor
Th	T-helper cell
Ti	Titanium
TIMP	Metallopeptidase inhibitor
TNF	Tumor necrosis factor
UNP	Unpolished
VEGF	Vascular endothelial growth factor
Zr	Zirconium
